# The Ability of Nuclease-Resistant RNA Aptamer against *Streptococcus suis* Serotype 2, Strain P1/7 to Reduce Biofilm Formation In Vitro

**DOI:** 10.3390/molecules27123894

**Published:** 2022-06-17

**Authors:** Apinyapat Matchawong, Chatchawan Srisawat, Sirikwan Sangboonruang, Chayada Sitthidet Tharinjaroen

**Affiliations:** 1 Division of Clinical Microbiology, Faculty of Associated Medical Sciences, Chiang Mai University, Chiang Mai 50200, Thailand; apinyapat_m@hotmail.com (A.M.); sirikwan.sang@cmu.ac.th (S.S.); 2 Department of Biochemistry, Faculty of Medicine, Siriraj Hospital, Mahidol University, Bangkok 10700, Thailand; chatchawan.sri@mahidol.ac.th; 3 Infectious Diseases Research Unit (IDRU), Faculty of Associated Medical Sciences, Chiang Mai University, Chiang Mai 50200, Thailand

**Keywords:** RNA aptamer, nuclease-resistant aptamer, *Streptococcus suis*, biofilm formation, *S. suis* infection

## Abstract

*Streptococcus suis*, a Gram-positive bacterium, is an important swine and human pathogen, with serotype 2 being the most prevalent strain found worldwide. Deafness, meningitis, and death (in severe cases) are observed in *S. suis*-infected cases. Development of the ligands that can bind to *S. suis* with high affinity and specificity could be beneficial for the diagnosis and treatment of *S. suis* infection. Herein, the nuclease-resistant RNA aptamers based on 2′-fluoropyrimidine modification against *S. suis* serotype 2, strain P1/7, were established using the cell- Systematic Evolution of Ligands by Exponential enrichment (SELEX) technique. One of the aptamers, R8-su12, could bind to the *S. suis* target strain as well as other *S. suis* serotypes, i.e., 1, 1/2, 9, and 14, but not to other bacteria tested, i.e., *S. pneumoniae* ATCC 49619, *Staphylococcus aureus* ATCC 25923, *Escherichia coli* ATCC 25922, and *Pseudomonas aeruginosa* ATCC 27853. Moreover, the R8-su12 RNA aptamer was also capable of inhibiting the biofilm formation of the *S. suis* target strain, making it potentially useful for the study of biofilm formation and the treatment of *S. suis* infection in humans and pigs in the future.

## 1. Introduction

*Streptococcus suis* is a zoonotic pathogen causing infectious diseases in pigs and humans such as meningitis, pericarditis, and septic shock [1,2]. To date, *S. suis* has been classified into 29 serotypes (SS) based on the specific antigens that appear on the polysaccharide capsule [3,4]. *S. suis* SS 2 infection was the most prevalent human case in Thailand, especially in the northern region [5,6]. Conventional methods for the identification of *S. suis* are culture and biochemical tests [7], which are time-consuming and sometimes yield controversial results as other bacteria such as *S. pneumoniae*, *S. bovis*, enterococci, viridans streptococci or even *Listeria monocytogenes* [8]. This can lead to a delayed or incorrect diagnosis. One of the important virulence characteristics of *S. suis* is biofilm formation [9,10], which allows for the bacteria to colonize permanently, increases resistance to the host immune system and antibiotics, and promotes their survival and proliferation [11]. An understanding of the biofilm formation involved in *S. suis* pathogenesis is needed for the effective control of *S. suis* infection in humans and animals.

Different types of ligands could be generated to bind *S. suis* with high affinity and specificity for research, diagnostic, and therapeutic purposes. One example is aptamers, which are single-stranded DNA or RNA [12]. The aptamers are generated using the technique called “Systematic Evolution of Ligands by EXponential enrichment” or SELEX, originally established in 1990 by Ellington and Tuerk [13,14]. In this study, the *S. suis*-specific RNA aptamers were developed because RNA has a greater ability to fold into more diverse three-dimensional conformations, which are capable of binding to target molecules, compared with DNA [15,16]. In addition, the RNA aptamers can be modified to become more resistant to the nucleases that could interfere with their functions. One of the modification methods is the substitution of the 2′-hydroxyl group of ribose sugar with the fluoro (-F), amino (-NH_2_), or methoxy (-OMe) groups [16,17]. 

In this study, nuclease-resistant RNA aptamers against *S. suis* SS 2, type strain P1/7 were successfully generated using the whole-cell SELEX approach. One of them, the R8-su12 RNA aptamer, could specifically recognize *S. suis* and inhibit biofilm formation, making it potentially useful for the study of biofilm formation and the treatment of *S. suis* infections in humans and pigs in the future.

## 2. Results

### 2.1. Nuclease-Resistant RNA Aptamer Selection Based on the Cell-SELEX Technique

**Figure 1:**
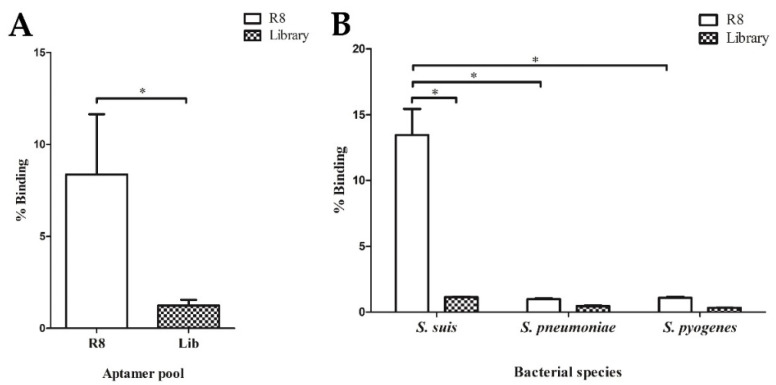
Characterization of RNA aptamer pool. (**A**) Enrichment of RNA aptamer pool derived from round eighth (R8) of cell-SELEX assay. The % binding of R8 RNA aptamer pool compared to the library (Lib) was presented. (**B**) Specificity of R8 RNA aptamer pool. The R8 RNA aptamer pool and random RNA Lib were tested with *S. suis*, *S. pneumoniae*, and *S. pyogenes*. * indicates the statistically significant difference using a Mann–Whitney U test (*p* < 0.05).

To select the nuclease-resistant RNA aptamers specific to *S. suis* SS 2, P1/7, approximately 10^14^ random RNA library pool was produced. A total of 10 mg/mL baker’s yeast tRNA was also added to the library pool as a binding competitor to reduce non-specific RNA binding. Then, the random RNA library mixture was incubated with 10^7^ live *S. suis* SS 2, P1/7 cells. The cell-SELEX was performed with a total of eight rounds. The enrichment of aptamer pool during selection rounds 4 (R4), 6 (R6), and 8 (R8) were measured using real-time reverse transcription (RT) qPCR compared with the random RNA library (Lib). The fold change in the relative binding affinity of RNA aptamer was increased in the later round of cell-SELEX (data not shown). The latest R8 RNA aptamer pool was used to determine the relative binding affinity compared to the starting N40 Lib. After calculation, the % binding of R8 RNA aptamer pool was higher than % binding of Lib with statistical significance (*p* = 0.023), implying the enrichment of the R8 RNA aptamer pool against *S. suis* SS 2, P1/7 (Figure 1A). The 8th round cell-SELEX pool was also screened for specificity using target and non-target cells, which were *S. pneumoniae* and *S. pyogenes*. The R8 RNA aptamer pool tested with *S. suis* SS 2, P1/7 showed the highest % binding when compared to the testing against *S. pneumoniae* and *S. pyogenes*, with statistical significance (*p* = 0.034 and *p* = 0.034, respectively) (Figure 1B). These results suggested that the R8 RNA aptamer pool was enriched and able to bind to *S. suis* SS 2, P1/7.

Therefore, the aptamer pool from R8 was cloned and sequenced. According to the sequencing analysis of 26 clones, they can be classified into four distinct groups as shown in Table 1. In the first group contained seven clones of short 40-nt long, calculated as 26.9%. The second group was also 40-nt long, with two corresponding sequences (7.7%). The third group consisted of four clones (15.4%), which were 39-nt long. The last group, named “ungrouped”, comprised of 13 different clones with various sequences, counting as 50% of the total number of all sequences (Table S1, Supplementary Materials).

**Figure 2:**
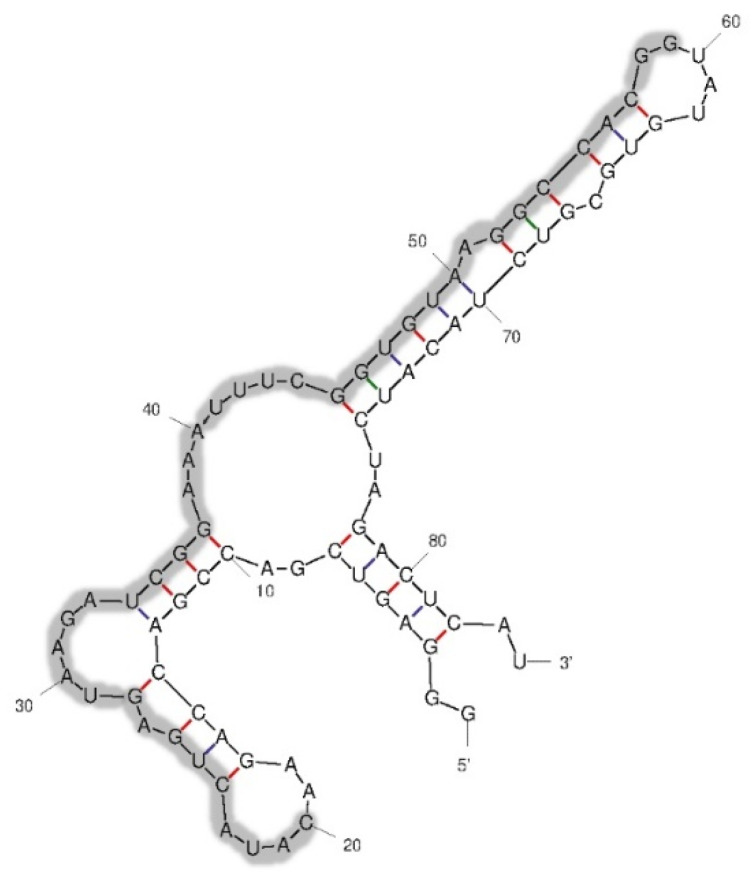
Predicted secondary structure of the R8-su12 RNA aptamer. The randomized region is 40-nt long and shown in the shaded area.

Thus, group 1 showed the highest consensus sequences frequency among the 26 clones, suggesting that they might have a high affinity toward *S. suis* SS2, P 1/7. The representative R8-su12 RNA aptamer from group 1 was then selected for the prediction of secondary structures; the folding temperature was set at 37 °C. The R8-su12 RNA aptamer had one predicted secondary structure with a folding energy (initial ΔG) equal to −24.30 kcal/mol. The predicted secondary structure consists of an external loop with four bases and one closing helices. Moreover, loops and stems were also found, as shown in Figure 2. Hence, the R8-su12 RNA aptamer was chosen for further characterization.

### 2.2. Specificity of R8-su12 RNA Aptamer

**Figure 3:**
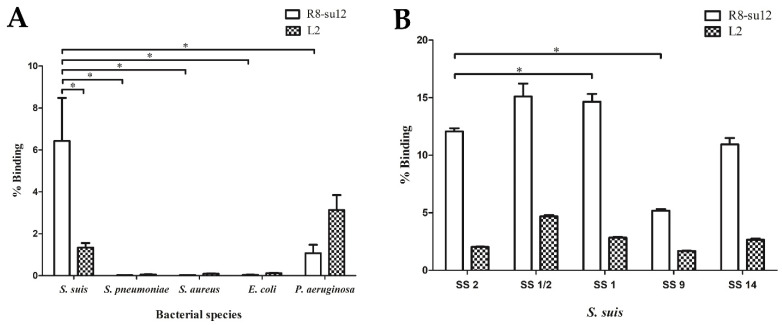
Specificity of the R8-su12 RNA aptamer. (**A**) The specificity of R8-su12 RNA aptamer was tested using target *S. suis* SS 2, P1/7 and non-target cells. (**B**) The specificity of R8-su12 RNA aptamer was tested using target *S. suis* SS 2, P1/7 and other *S. suis* serotypes. * indicates a statistically significant difference using a Mann–Whitney U test (*p* < 0.05).

To test the specificity of the R8-su12 RNA aptamer, the target *S. suis* SS 2, P1/7 and non-target cells including the American Type Culture Collection (ATCC) strains of *S. pneumoniae*, *S. aureus*, *E. coli*, and *P. aeruginosa*, were evaluated. The negative control—L2 RNA aptamer (5′-GGGAGUCGACCGACCAGAAGAAGCUGCUUCAAAAUAGAUCUACACUCACAUAAGGCGAAUAUGUGCGUCUACAUCUAGACUCAU-3′) was used in this experiment, instead of a random RNA Lib. The R8-su12 and L2 RNA aptamer were incubated with target and non-target cells. As shown in Figure 3A, the R8-su12 RNA aptamer show statistically significant higher binding affinities to *S. suis* SS 2, P1/7 when compared with *S. pneumoniae*, *S. aureus*, *E. coli*, and *P. aeruginosa*. (*p* = 0.021, 0.020, 0.020, and 0.021, respectively). 

Moreover, the R8-su12 RNA aptamer was also tested with *S. suis* SS 1/2, 1, 9, and 14. These strains are closely related to the target cells and showed a high prevalence of *S. suis* infection strains in Thailand [18]. The R8-su12 RNA aptamer showed an ability to bind to these *S. suis* strains. Interestingly, the R8-su12 RNA aptamer showed the highest % binding when tested with *S. suis* SS 1/2, followed by *S. suis* SS 1, 2, 14, and 9, respectively (Figure 3B). In addition, the R8-su12 RNA aptamer showed a significant higher binding affinity against *S. suis* SS 1 (*p* = 0.021) compared to the target cells. On the other hand, a lower binding affinity against *S. suis* SS 9 (*p* = 0.021) was shown. No significant binding was found in *S. suis* SS 1/2 (*p* = 0.275) and *S. suis* SS 14 (*p* = 0.127) (Figure 3B).

### 2.3. The Effect of R8-su12 RNA Aptamer on S. suis SS 2, P1/7 Biofilm Formation

**Figure 4:**
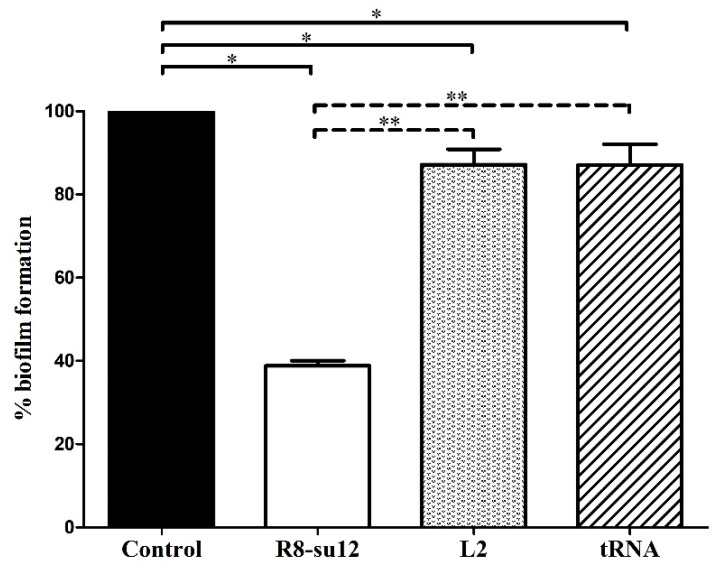
Reduction in biofilm formation by R8-su12 RNA aptamer. The *S. suis* SS 2, P1/7 was cultured in BHI broth with supplements (control) and in the presence of R8-su12 RNA aptamer (R8-su12), L2 RNA aptamer (L2), and baker’s yeast tRNA (tRNA). After 3 days of incubation without shaking, production of biofilm was determined using crystal violet biofilm assay. Percentage of *S. suis* SS 2, P1/7 biofilm formation was calculated. * indicates *p* < 0.05 and ** indicates *p* < 0.001 using the one-way ANOVA test.

Biofilm formation is associated with the virulence of *S. suis* [11] and one biofilm-production-related molecule—polysaccharide intercellular adhesin (PIA) is located on the cell surface [19]. Hence, we hypothesized that the derived aptamer that targeted the surface molecules on *S. suis* cells might affect the biofilm formation. As shown in the result, the biofilm formation of *S. suis* SS 2, P1/7 cultured with R8-su12 RNA aptamer, L2 RNA aptamer and baker’s yeast tRNA was significantly reduced by 61.2%, 12.8% and 12.9 %, respectively, when compared to control (*p* < 0.05). Noticeably, the influential activity that decreased biofilm formation was also observed in R8-su12 RNA aptamer treated *S. suis* SS 2, P1/7, showing 55.5% and 55.4% reductions when compared to L2 RNA aptamer (*p* < 0.001) and baker’s yeast tRNA (*p* < 0.001), respectively. While the presence of L2 RNA aptamer and baker’s yeast tRNA in the culture medium, biofilm formation showed no statistically significant difference (*p* = 0.988) (Figure 4). These results implied that the R8-su12 RNA aptamer can reduce the production of *S. suis* SS 2, P1/7 biofilm formation.

## 3. Discussion

*S. suis* is reported as a zoonotic pathogen causing infectious diseases in pigs and humans, such as meningitis, pericarditis, and septic shock [1,2]. *S. suis* serotype 2 infection is the most prevalent human case in the world, including Thailand, especially in the northern region [5,6]. Nowadays, no vaccine is available and rapid disease progression is of great concern. Thus, the prevention and control of *S. suis* infection is needed. The development of ligands that can bind to *S. suis* with high affinity and specificity could be beneficial for the diagnosis and treatment of *S. suis* infection. 

In this study, the nuclease-resistant RNA aptamer, R8-su12, against *S. suis* SS 2, P1/7 was first established. RNA aptamers were chosen over DNA aptamers for their ability to fold into a more diverse, three-dimensional structures [15,16]. Moreover, they can be generated with 2′-fluoropyrimidines (2′-fluoro-dCTP and 2′-fluoro-dUTP) substitution to improve their stability and resistance to nucleases [20,21,22]. The whole cell-SELEX approach was used because the identification and production of unique proteins as selection targets are difficult, as the molecules on the *S. suis* cell surface show high levels of similarity [23]. This approach would also help to generate the aptamers that target multiple surface antigens, and would not require the isolation and purification of target proteins, thus avoiding the misfolding or denaturation of the target molecules [24]. In addition, we developed the electrophoretic separation technique to partition non-binding RNAs from the aptamers that bound to bacterial cells, and this was used in the last two rounds of selection. Using this technique, non- or weak-binding RNAs were actively separated into the agarose gel under an electric field due to their negative charges, while the aptamers bound with high affinity to *S. suis* remained in the sample well with bacterial cells. Theoretically, the active separation using electrophoresis would remove non-binding RNAs from the target cells more efficiently than the washing separation, which relies on the passive dissociation of RNAs. Therefore, the stringency of selection was increased, resulting in the successful recovery of the high-affinity *S. suis* aptamers.

For the specificity determination, the data showed that the R8-su12 RNA aptamer bound to target cells, *S. suis* SS 2, P1/7, as well as the others *S. suis* serotypes, 1, 1/2, 2, and 14. Unfortunately, the target molecule of the aptamer was not determined in this study. We speculated that the potential targets of the R8-su12 RNA aptamer could be some components of capsular polysaccharides (CPSs) that are shared between these serotypes. There are several studies revealed that *S. suis* serotypes 1, 1/2, 2, and 14 contain sialic acid in their CPSs [9,25,26]. Therefore, this might be one of the candidate molecules to which R8-su12 RNA aptamer can bind. However, it would be helpful to identify the potential targets of this aptamer in further studies. 

One of the important virulence characteristics of *S. suis* is biofilm formation. This leads to an increased resistance to host defenses and antimicrobial agents, making pathogens difficult or impossible to eliminate [10]. Therefore, the R8-su12 RNA aptamer was further tested for its effects on *S. suis* biofilm formation. We found that the derived aptamer can reduce the biofilm formation of *S. suis* SS 2, P1/7. The studies on the bacteria biofilm revealed that the regulatory mechanism can be divided into eight categories, depending on the different communication signaling molecules produced by bacteria [20]. These consist of: (1) bacterial death and dissolution mechanism, which plays an important role in the formation and development of bacterial biofilms; (2) extracellular polymeric substance matrix (EPS) that consists of some extracellular polysaccharides, DNA, proteins, and some other macromolecules; (3) two-component systems (TCS), which mainly involve receptor histidine protein kinases (HPK) and response regulators (RR); (4) extracytoplasmic function (ECF) signaling pathway that regulate the production of alginate, which is important for the biofilm structure; (5) intracellular second messenger cyclic diguanylate (c-di-GMP), which can induce the production of extracellular polymeric substances (EPS) and surface adhesins, leading to the formation of bacterial biofilms; (6) small RNAs (sRNAs), which are involved in the regulation of expression or activity of important transcriptional regulators and components required for cell attachment and biofilm formation; (7) bacterial biofilm intracellular signal transduction system called bacterial quorum sensing (QS) system; (8) the polysaccharide intercellular adhesin (PIA) which plays an important role in the bacterial aggregation stage of bacterial biofilm formation. Moreover, the study by Zhou [27] revealed that the reduction in O-acetylserine (thiol)-lyase B (CysM), a key enzymatic regulator of cysteine synthesis, can inhibit *S. suis* biofilm formation. The study also showed that the ability of the complementary mutant (CΔcysM) strain to form a biofilm may indirectly related to the QS system. It is possible that the R8-su12 RNA aptamer might bind to those molecules, indirectly or directly playing a role in biofilm formation. However, additional studies are required to investigate how the aptamer affects biofilm formation.

## 4. Materials and Methods

### 4.1. Bacteria Used and Culture Condition

The *Streptococcus suis* serotype 2, type strain P1/7 (*S. suis* SS 2, P1/7) (kindly provided by Division of Bacteriology, Department of Microbiology, Faculty of Medicine, Chiang Mai University) was used as the target cell in this study. For specificity testing, other bacterial species, including *Escherichia coli* ATCC 25922, *Pseudomonas aeruginosa* ATCC 27853, *Staphylococcus aureus* ATCC 25923, *Streptococcus pneumoniae* ATCC 49619, *S. pyogenes* ATCC 19615, *S. suis* serotype 1 DMST, *S. suis* serotype 1/2 DMST, *S. suis* serotype 14 DMST, and *S. suis* serotype 9 clinical isolates were used. *S. suis* SS 2, P1/7 was cultured onto 5% blood agar (BA) (Becton Dickinson, NJ, USA) and incubated at 37 °C, 5% CO_2_ for 18–24 h. For other bacterial species were incubated overnight for 18–24 h at 37 °C, 5% CO_2_ on 5% BA (Becton Dickinson, NJ, USA) or trypticase soy agar (Becton Dickinson, NJ, USA) depending on their optimal medium. To prepare the target cells for the SELEX process, fresh colonies from BA (Becton Dickinson, NJ, USA) were harvested and washed thrice with 1 mL of Phosphate Buffer Saline (PBS). Then, the bacterial cells were adjusted to reach at the desired OD_600_ with a total of 1 mL of binding buffer before being loaded into a centrifugal filter (Ultrafree^®^-MC with Microporous Durapore^®^ PVDF Membrane, pore size 0.45 µm, Merck, NJ, USA).

### 4.2. Cell-SELEX Technique

The starting 2′-F pyrimidine modified RNA Lib was prepared by in vitro transcription of a N40 DNA Lib containing a 40-nt random region and primer binding sites (5′-AGTAATACGACTCACTATAGGGAGTCGACCGACCAGAA-N40-TATGTGCGTCTACATCTAGACTCAT-3′) using T7 R&D polymerase (50 U/µL) (Lucigen Corporation, WI, USA).

To obtain the practical diversity of the Lib about 10^14^ molecules, 100 pmol of random RNA Lib was used in the first round of cell-SELEX. The 2′-F random RNA Lib was thermally equilibrated for proper folding in a total volume of 100 µL binding buffer (0.05 M HEPES pH 7.4, 0.1 M NaCl, and 0.01 M MgCl_2_) with 10 mg/mL baker’s yeast tRNA (Merck, NJ, USA) at 65 °C, followed by a cool-down at room temperature for 5 min. The freshly prepared 10^7^ cells of *S. suis* SS 2, P1/7 were incubated with the equilibrated random RNA Lib at room temperature in the centrifugal filter for 60 min in rounds 1–7 and 50 min in round 8, with gentle rotation. The unbound or weakly bound aptamers were partitioned by washing in binding buffer for various specific volumes, numbers of washes, and incubation times on the rotator according to the selection protocol (Table 2). For rounds 7 and 8, the separation was performed using an electrophoresis technique to increase the selection stringency. In brief, after the binding step, the bacterial pellet was washed once with 100 µL of binding buffer for 1 min, and then resuspended with 50 µL of binding buffer. The suspension was transferred into a well of agarose gel (3% in 0.5× TAE). After that, 0.5× TAE was carefully added into an electrophoresis chamber. Gel electrophoresis was run using 100 V for 10 min. The target cells with bound aptamers were collected and washed thrice with 100 µL of binding buffer for 1 min each time before the elution step. 

The bound RNAs were eluted using 100 µL of elution buffer (8 M urea, 5 mM EDTA pH 8.0), followed by ethanol precipitation. The RNA pool was reverse-transcribed using Superscript^®^ III Reverse Transcriptase (Thermo Fisher Scientific, Waltham, MA, USA) and 3′-N40 primer (5′-ATGAGTCTA GATGTAGACGCACATA-3′). Then, the cDNA was amplified with *Taq* DNA polymerase (Thermo Fisher Scientific, Waltham, MA, USA) using 3′-N40 primer and 5′-primer containing T7 promoter (underlined) (5′-AGTAATACGACTCACTATAGGGAGTCGACCGACCAGAA-3′). The PCR products were transcribed in vitro, purified using the Qiaquick PCR Purification Kit (Qiagen, Hilden, Germany), and used for the next round of cell-SELEX. The cell-SELEX was performed until the 8th round (R8).

### 4.3. Evaluation of Relative Binding Affinity and Specificity of the RNA Aptamer 

The enrichment of the aptamer pool in selected rounds (Rx), which were R4, R6, and R8, was quantified by relative binding affinity using the binding assay. One hundred picomoles of either the control RNA Lib or selected aptamer pool in the presence of baker’s yeast tRNA was incubated with approximately 10^7^ cells of *S. suis* SS 2, P1/7. Both Lib and Rx were saved for approximately 10 µL as input RNA. After binding, washing, and elution steps, as described above, the amount of input and bound RNAs were quantified using real-time RT qPCR. The real-time master mix was generated in a total 10 µL per reaction comprising the final concentration of 1× SensiFAST^™^ SYBR^®^ No-ROX Kit (Meridian Bioscience, TN, USA), 0.5 µM of each 3′-N40 primer and 5′-primer (5′-GGGAGTCGACCGACCAGAA-3′), and 0.5 µL of cDNA from each RT reaction (Lib input, Lib elute, Rx input, Rx elute, NTC). Each sample was performed in triplicate. For non-template control (NTC), real-time PCR master mix and cDNA from NTC-RT reaction were used. The real-time RT qPCR was performed under the following conditions: 94 °C for 30 s, 60 °C for 30 s, 72 °C for 30 s, with a total of 45 cycles. The fluorescence signal was read at 60 °C and the association curve was set at 65 °C to 95 °C. The binding percentage was calculated using Formula (1):

% binding = 2 − (Ct_elute_ − Ct_input_), (1)

where Ct_elute_ refers to the cycle threshold of elute sample and Ct_input_ refers to the cycle threshold of input sample. The % binding of random RNA library (% binding_Lib_) and the % binding of Rx (% binding_Rx_) were then calculated into the fold change using Formula (2):

Fold change = % binding_Rx_/% binding_Lib_, (2)

For specificity testing, the R8 aptamer pool and representative R8-su12 RNA aptamers were screened toward *S. suis* SS 2, P1/7 and non-target cells, including other species of *Streptococcus* (*S. pyogenes, S. pneumoniae*), Gram-positive bacteria (*S. aureus*), Gram-negative bacteria (*E. coli*, *P. aeruginosa*), and other *S. suis* serotype (*S. suis* SS1/2, 1, 9, and 14). One hundred picomole of the control (random RNA Lib pool or L2 aptamer) and the derived nuclease-resistant RNA aptamers were incubated with 10^7^ of target cells, *S. suis* SS 2, P1/7 and non-target cells. Then, the cell-SELEX was processed until the eluate was obtained. After that, it was used as a template in the binding assay using real-time RT qPCR. The binding percentage and fold change were calculated as described above.

### 4.4. Aptamer Characterization

The aptamer pool from cell-SELEX round 8 was cloned using pTG19-T PCR cloning vector B (Vivantis, Selangor, Malaysia). Twenty-six clones were sequenced and analyzed for similarity using the Clustal Omega Multiple Sequence Alignment (https://www.ebi.ac.uk/Tools/msa/clustalo/ accessed on 11 November 2020). The selected RNA aptamer was also predicted for secondary structure using the mfold Web Server (http://www.unafold.org/mfold/applications/rna-folding-form.php accessed on 7 February 2021) to determine the binding sites between the aptamer and target cells.

### 4.5. Biofilm Formation Assay

To determine the effect of the RNA aptamer on the biofilm formation of *S. suis* SS 2, P1/7, the selected R8-su12 RNA aptamer was tested. The determination of *S. suis* SS 2, P1/7 biofilm formation was preliminarily tested based on the crystal violet biofilm assay [28]. 

To examine the RNA aptamer’s ability to inhibit biofilm formation, the detection of biofilm formation was carried out according to Meng et al. [28] with some modifications. In brief, 200 μL of *S. suis* SS 2, P1/7 from the overnight culture at 37 °C was mixed with 100 μL of 4.76 ng/µL (176 nM) of R8-su12 RNA aptamer with 10 mg/mL baker’s yeast tRNA, negative control L2 RNA aptamer with 10 mg/mL baker’s yeast tRNA, and 10 mg/mL baker’s yeast tRNA alone. Then, the mixture was transferred into a 24-well polystyrene plate containing 1.8 mL of BHI broth. The plates were incubated at 37 °C for 3 days without shaking. After staining with 0.1% crystal violet for 30 min, the OD_600_ was measured. The BHI broth with the supplements was performed as a negative control, and its OD_600_ was set as blank. These blank absorbance values and cut off values (ODc) were used to calculate and interpret the biofilm formation result as described [28]. Each sample in the biofilm formation assay was performed in quadruplicate in three independent experiments.

### 4.6. Statistically Analysis

All samples were tested in triplicate with at least three independent experiments. The one-way analysis of variance and Mann–Whitney U test were used to determine the statistically significant when *p* < 0.05 or 0.001. All the calculations were performed using the SPSS Statistics 6.0 software (IBM Corp., Armonk, NY, USA).

## 5. Conclusions

Herein, the nuclease resistant RNA aptamer specific to *S. suis* SS 2, strain P1/7, R8-su12, was successfully constructed. Moreover, the derived RNA aptamer revealed the ability to bind to other pathogenic *S. suis* (serotype 1/2, 1, 9, and 14). Remarkably, the R8-su12 RNA aptamer can significantly reduce the biofilm formation, one of the *S. suis* virulence factors. However, the target ligand of the aptamer on the *S. suis* SS 2, strain P1/7 cell surface would be further identified. This might be useful for understanding the pathogenesis, leading to an early diagnostic, treatment, and effective control of *S. suis* infection.

## Figures and Tables

**Table 1:** Summarization of consensus randomized sequences of the RNA R8 aptamer pool.

Group	Representative Clone	Consensus Randomized Sequences	Nucleotides	Frequency (%)
1	R8-su12	CAUACUGAGUAAGAUCGGAAAUUUCGGUGUAAGGCCACGG	40	7/26 (26.9%)
2	R8-su057	UGGAUGUAUGGAACUUGCAGAUCUUAACUGCACGAAGCGU	40	2/26 (7.7%)
3	R8-su15	ACACGUUGCUGAAACAUACCGAGUAACAUAAAGCGGGUG	39	4/26 (15.4%)
4	Ungrouped	Different clones with various sequences	40	13/26 (50%)

**Table 2:** The aptamer selection protocol for cell-SELEX using *S. suis* SS 2, P1/7 as target cells.

SELEX Round	Input RNA (pmol)	*S. suis* SS 2, P1/7 (Cells)	Washing
1	100	1.03 × 10^7^	100 µL × 5 times, 1 min each
2	50	1.02 × 10^7^	100 µL × 5 times, 1 min each
3	50	1.00 × 10^7^	100 µL × 5 times, 3 min each
4	25	1.11 × 10^7^	100 µL × 5 times, 3 min each
5	10	1.21 × 10^7^	100 µL × 5 times, 3 min each
6	10	1.29 × 10^7^	100 µL × 5 times, 5 min each
7	10	1.01 × 10^7^	100 µL for 1 min, followed by electrophoretic separation
8	10	1.07 × 10^7^	100 µL for 1 min, followed by electrophoretic separation

## Data Availability

Not applicable.

## Supplementary Materials

The following supporting information can be downloaded at: https://www.mdpi.com/article/10.3390/molecules27123894/s1, Table S1: The sequencing analysis of the RNA R8 aptamer pool.

## Sample Availability

Samples of the compounds are not available from the authors.

## References

[1] Staats J.J., Feder I., Okwumabua O., Chengappa M.M. (1997). *Streptococcus Suis*: Past and present. Vet. Res. Commun..

[2] Tang J., Wang C., Feng Y., Yang W., Song H., Chen Z., Yu H., Pan X., Zhou X., Wang H. (2006). Streptococcal toxic shock syndrome caused by *Streptococcus suis* serotype 2. PLoS Med..

[3] Okura M., Osaki M., Nomoto R., Arai S., Osawa R., Sekizaki T., Takamatsu D. (2016). Current taxonomical situation of *Streptococcus suis*. Pathogens.

[4] Dutkiewicz J., Sroka J., Zając V., Wasiński B., Cisak E., Sawczyn A., Kloc A., Wójcik-Fatla A. (2017). *Streptococcus suis*: A re-emerging pathogen associated with occupational exposure to pigs or pork products. part I—epidemiology. Ann. Agric. Environ. Med..

[5] Wangkaew S., Chaiwarith R., Tharavichitkul P., Supparatpinyo K. (2006). *Streptococcus suis* infection: A series of 41 cases from Chiang Mai University Hospital. J. Infect..

[6] Kerdsin A., Dejsirilert S., Puangpatra P., Sripakdee S., Chumla K., Boonkerd N., Polwichai P., Tanimura S., Takeuchi D., Nakayama T. (2011). Genotypic profile of *Streptococcus suis* serotype 2 and clinical features of infection in humans, Thailand. Emerg. Infect. Dis..

[7] Hommez J., Devriese L.A., Henrichsen J., Castryck F. (1986). Identification and characterization of *Streptococcus suis*. Vet. Microbiol..

[8] Goyette-Desjardins G., Auger J.P., Xu J., Segura M., Gottschalk M. (2014). *Streptococcus suis*, an important pig pathogen and emerging zoonotic agent—An update on the worldwide distribution based on serotyping and sequence typing. Emerg. Microbes. Infect..

[9] Van Calsteren M.R., Gagnon F., Lacouture S., Fittipaldi N., Gottschalk M. (2010). Structure determination of *Streptococcus suis* serotype 2 capsular polysaccharide. Biochem. Cell. Biol..

[10] Donlan R.M., Costerton J.W. (2002). Biofilms: Survival mechanisms of clinically relevant microorganisms. Clin. Microbiol. Rev..

[11] Grenier D., Grignon L., Gottschalk M. (2009). Characterisation of biofilm formation by a *Streptococcus suis* meningitis isolate. Vet. J..

[12] Stoltenburg R., Reinemann C., Strehlitz B. (2007). SELEX—A (r)evolutionary method to generate high-affinity nucleic acid ligands. Biomol. Eng..

[13] Ellington A.D., Szostak J.W. (1990). In vitro selection of RNA molecules that bind specific ligands. Nature.

[14] Tuerk C., Gold L. (1990). Systematic evolution of ligands by exponential enrichment: RNA ligands to bacteriophage T4 DNA polymerase. Science.

[15] Darmostuk M., Rimpelova S., Gbelcova H., Ruml T. (2015). Current approaches in SELEX: An update to aptamer selection technology. Biotechnol. Adv..

[16] Shigdar S., Macdonald J., O’Connor M., Wang T., Xiang D., Al Shamaileh H., Qiao L., Wei M., Zhou S.F., Zhu Y. (2013). Aptamers as theranostic agents: Modifications, serum stability and functionalisation. Sensors.

[17] Sampson T. (2003). Aptamers and SELEX: The technology. World Pat. Inf..

[18] Thongkamkoon P., Kiatyingangsulee T., Gottschalk M. (2017). Serotypes of *Streptococcus suis* isolated from healthy pigs in Phayao Province, Thailand. BMC Res. Notes.

[19] Wang Y., Wang Y., Sun L., Grenier D., Yi L. (2018). *Streptococcus suis* biofilm: Regulation, drug-resistance mechanisms, and disinfection strategies. Appl. Microbiol. Biotechnol..

[20] Eaton B.E., Pieken W.A. (1995). Ribonucleosides and RNA. Annu. Rev. Biochem..

[21] Pieken W., Tasset D., Janjic N., Gold L., Kirschenheuter G.P. (1997). High Affinity Nucleic Acid Ligand Containing Modified Nucleotides. U.S. Patent.

[22] Polisky B., Jenison R.D., Gold L. (1996). High-Affinity Nucleic Acid Ligands That Discriminate between Theophylline and Caffeine. U.S. Patent.

[23] Baums C.G., Valentin-Weigand P. (2009). Surface-associated and secreted factors of *Streptococcus suis* in epidemiology, pathogenesis and vaccine development. Anim. Health Res. Rev..

[24] Nosaz Z., Rasoulinejad S., Mousavi Gargari S.L. (2020). Development of a DNA aptamer to detect *Brucella abortus* and *Brucella melitensis* through cell SELEX. Iran. J. Vet. Res..

[25] Van Calsteren M.R., Gagnon F., Calzas C., Goyette-Desjardins G., Okura M., Takamatsu D., Gottschalk M., Segura M. (2013). Structure determination of *Streptococcus suis* serotype 14 capsular polysaccharide. Biochem. Cell. Biol..

[26] Van Calsteren M.R., Goyette-Desjardins G., Gagnon F., Okura M., Takamatsu D., Roy R., Gottschalk M., Segura M. (2016). Explaining the Serological Characteristics of *Streptococcus suis* Serotypes 1 and 1/2 from Their Capsular Polysaccharide Structure and Biosynthesis. J. Biol. Chem..

[27] Zhou Y., Yu F., Chen M., Zhang Y., Qu Q., Wei Y., Xie C., Wu T., Liu Y., Zhang Z. (2022). Tylosin Inhibits *Streptococcus suis* Biofilm Formation by Interacting With the O-acetylserine (thiol)-lyase B CysM. Front. Vet. Sci..

[28] Meng X., Shi Y., Ji W., Meng X., Zhang J., Wang H., Lu C., Sun J., Yan Y. (2011). Application of a bacteriophage lysin to disrupt biofilms formed by the animal pathogen *Streptococcus suis*. Appl. Environ. Microbiol..

