# Supplementary material for: The Ability of Nuclease-Resistant RNA Aptamer against Streptococcus suis Serotype 2, Strain P1/7 to Reduce Biofilm Formation In Vitro
Source: Molecules. 2022 Jun 17;27(12):3894. doi: 10.3390/molecules27123894 (PMC9228048; doi:10.3390/molecules27123894)
Supplement: Supplementary file 1 [file molecules-27-03894-s001.zip › molecules-1740655-supplementary.pdf]

Supplementary Materials

Table S1: The sequencing analysis of the RNA R8 aptamer pool.

GROUP 1 [7/26] [26.9 %]

R8-su08	CAUACUGAGUAAGAUCGGAAAUUUCGGUGUAAGGCCACGG
R8-su11	CAUACUGAGUAAGAUCGGAAAUUUCGGUGUAAGGCCACGG
R8-su12	CAUACUGAGUAAGAUCGGAAAUUUCGGUGUAAGGCCACGG
R8-su13	CAUACUGAGUAAGAUCGGAAAUUUCGGUGUAAGGCCACGG
R8-su027	CAUACUGAGUAAGAUCGGAAAUUUCGGUGUAAGGCCACGG
R8-su101	CAUACUGAGUAAGAUCGGAAAUUUCGGUGUAAGGCCACGG
R8-su215	CAUACUGAGUAAGAUCGGAAAUUUCGGUGUAAGGCCACGG

GROUP 2 [2/26] [7.7 %]

R8-su061	UGGAUGUAUGGAACUUGCAGAUCUUAACUGCACGAAGCGU
R8-su057	UGGAUGUAUGGAACUUGCAGAUCUUAACUGCACGAAGCGU

Group 3 [4/26] [15.4 %]

R8-su15	ACACGUUGCUGAAACAUACCGAGUAACAUAAAGCGGGUG
R8-su16	ACACGUUGCUGAAACAUACCGAGUAACAUAAAGCGGGUG
R8-su007	ACACGUUGCUGAAACAUACCGAGUAACAUAAAGCGGGUG
R8-su011	ACACGUUGCUGAAACAUACCGAGUAACAUAAAGCGGGUG

UNGROUPED [13/26] [50 %]

R8-su014	AAAUAGACUAUUCCGGUGUAGUAGCCAGUAGAGACAUGAG
R8-su037	AAAUCAAGGGAAGAAGCCACAGUUUUUAUGCCAUCGGGGA
R8-su147	AAAUACCGAUAUCGUGCCACAUACUGAGAAACACUGUCGG
R8-su137	AUAUCGAGAUCUUGUACUUGUGAUAUGGUGUUAUGGGGUG
R8-su024	CAUACUGAGAAAGAUCGGUCAAGAGUAGUGGAAUUCUGCC
R8-su073	CAUAUGGGACCGACCAUUUUACAUUGAUAAGCUGUACGGU
R8-su209	CCACGUGAAGGAGAAAGUAACAAGGUUGUUUGUGUCGCUG
R8-su068	GUUAGUCGGUUUCUAACAAAACGAUGUUUGAGAGGUGGCC
R8-su012	UAUGUGUACAUGCACUAUUAACCGUACUGAUAUUCGUGUU
R8-su36	UAUUGGGUUGAUCUACAUUGAGGUGUGACGUCGUGGAAGG
R8-su025	UUAACUGUGCAGAUUCAGUUAAUGUAGUAGAUGCCACCGG
R8-su031	UGAAACAUACGGAGAAACAUUUUGGAGUUGAAGGUCGAUU
R8-su164	UCGGUAAUUACGCCAUGUUCUUCGCUAGGAACACUAAGGG
